# Lithium chloride effectively kills the honey bee parasite *Varroa destructor* by a systemic mode of action

**DOI:** 10.1038/s41598-017-19137-5

**Published:** 2018-01-12

**Authors:** Bettina Ziegelmann, Elisabeth Abele, Stefan Hannus, Michaela Beitzinger, Stefan Berg, Peter Rosenkranz

**Affiliations:** 1 University of Hohenheim, Apicultural State Institute, 70593 Stuttgart, Germany; 2 siTOOLs Biotech GmbH, Lochhamerstrasse 29 A, 82152 Planegg, Germany; 3 Bayerische Landesanstalt für Weinbau und Gartenbau, Fachzentrum Bienen, An der Steige 15, 97209 Veitshöchheim, Germany

## Abstract

Honey bees are increasingly important in the pollination of crops and wild plants. Recent reports of the weakening and periodical high losses of managed honey bee colonies have alarmed beekeeper, farmers and scientists. Infestations with the ectoparasitic mite *Varroa destructor* in combination with its associated viruses have been identified as a crucial driver of these health problems. Although yearly treatments are required to prevent collapses of honey bee colonies, the number of effective acaricides is small and no new active compounds have been registered in the past 25 years. RNAi-based methods were proposed recently as a promising new tool. However, the application of these methods according to published protocols has led to a surprising discovery. Here, we show that the lithium chloride that was used to precipitate RNA and other lithium compounds is highly effective at killing *Varroa* mites when fed to host bees at low millimolar concentrations. Experiments with caged bees and brood-free artificial swarms consisting of a queen and several thousand bees clearly demonstrate the potential of lithium as miticidal agent with good tolerability in worker bees providing a promising basis for the development of an effective and easy-to-apply control method for mite treatment.

## Introduction

Honey bees play a central role in agriculture as pollinators and their global economic contribution to food production is estimated between 235 and 285 bn US$ annually^1^. Their value to the ecosystem lies in the fact that honey bees pollinate more than 90% of insect-pollinated plants, and as generalist, they are crucial for the buffering of pollination networks^2^. Therefore, recent reports of a general weakening of honey bees, which has led to periodical high losses of managed colonies have not only startled beekeeper and bee scientists but have also raised public concern. High colony losses not only exacerbate the management of honey bee colonies but also significantly increase the costs of pollination services^3^ with consequences to global crop production.

Although the reasons for the current problems of honey bee health have not been completely unravelled, the haemolymph-sucking ectoparasitic mite *Varroa destructor* is considered a crucial driver of this global plight of honey bee (*Apis mellifera*) colonies^4–7^, and no other parasite or pathogen has had a comparable impact on bee health or beekeeping in the long history of apiculture^8^. Originally, *V*. *destructor* exclusively parasitized the Eastern honey bee *Apis cerana*. In the new host, *A*. *mellifera* the mite population grows exponentially during the periods when the honey bee colony has brood because female mites can exclusively reproduce within sealed worker or drone brood cells. High mite infestation levels lead to severe host damages by loss of haemolymph and even worse by the transmission and activation of certain honey bee viruses^9,10^. The most striking difference between the transmission of viruses per feeding and by contact among bees within a colony and by *Varroa* is that the mite directly injects the virus into the haemolymph, leading to a by-pass of host defence mechanisms. Migratory beekeeping practices and high colony densities have further aggravated the problem by favouring the horizontal transmission of bee viruses among neighbouring colonies through the mite vector^11^. This scenario has resulted in an increased prevalence of more virulent strains of honey bee viruses^12^ with the consequence that the damage threshold for mite-infested honey bee colonies has decreased over the past 20 years^13^.

Under the conditions of the common beekeeping practice in non-tropical regions, which are characterized by high colony densities, prevention of swarming and periodical mite control, it seems difficult to achieve a more balanced host-parasite relationship^14^. Thus far, a long-term survival of *A*. *mellifera* colonies without any control measures is nearly exclusively reported from feral populations^15–17^ or colonies kept continuously under natural selection pressure^18,19^. In most cases, it is not even clear whether this survival is due to tolerance by limiting the damage to the host or rather to resistance mechanisms by reducing the reproductive success of the parasite^20^. Despite promising approaches in selective breeding for hygienic behaviour and/ or low mite reproduction^21,22^, it seems rather doubtful that tolerance breeding alone will solve the global *Varroa* problem in the foreseeable future. Therefore, nearly all currently managed bee hives require yearly treatments^8^ and it is very likely that this will be further necessary in the coming decades.

Considering this challenge for global apiculture, it is remarkable that all currently registered synthetic veterinary products for the control of Varroosis are based on only a few compounds, i.e. the organophosphate coumaphos, some pyrethroids and the formamidine amitraz. No new active compounds have been registered for more than 25 years^23^. As a consequence, *Varroa* mites have evolved resistance to all available synthetic acaricides^24,25^. The alternative use of organic acids and essential oils has unfortunately resulted in variable and inconsistent efficacy^8^. Despite increased attention for *Varroa* treatment periodic high colony losses have been reported in nearly all countries in Europe and North America^26–31^. This clearly demonstrates that more research activities on *Varroa* treatment are urgently needed to develop novel, more effective agents against Varroosis.

An actual new approach was reported recently by Garbian *et al*.^32^, who outlined the use of RNAi to control *Varroa* infestation. Briefly, double-stranded RNA (dsRNA) matching essential *Varroa* genes was fed to bees and horizontally transferred to parasitizing mites via ingested haemolymph. The approach elegantly uses the host organism as a vector to deliver the selective and lethal dsRNAs to the parasite. Indeed, the authors show that in *Varroa*, dsRNA knocks down the targeted genes and leads to mite loss of up to 60% within 2 months. Intrigued by this approach, we set out to improve the method with higher activity and a shorter treatment period by targeting a new selection of essential *Varroa* specific genes. However, an initial pilot study revealed unexpected results leading to a completely new approach to a systemic treatment of *Varroa* mites.

## Results and Discussion

### Pilot study: From RNAi to lithium chloride

In a pilot study, mite-infested honey bees were caged and fed sucrose syrup containing dsRNAs of potentially essential *Varroa* genes (Supplementary Table S1). Plain sucrose syrup (untreated) and syrup with dsRNA based on the coding sequence for the green fluorescent protein GFP (dsGFP ctrl) served as controls. GFP is expressed in the bioluminescent hydrozoan jellyfish *Aequorea victoria*. The GFP sequence was chosen as a control because no homologous gene exists in the genome of honey bees or the *Varroa* mite. In the untreated group mite mortality was <5%. In contrast, all mites on bees that received sucrose solution containing dsRNA targeting *Varroa* genes were effectively killed within three days. An identical effect on mites, however, was observed in a control experiment in which bees were fed GFP dsRNA (Supplementary Fig. S1). These results ruled out the proposed RNAi mediated mechanism but suggested either a yet unknown effect of RNA or the activity of other components in the test solution. As high concentrations of lithium chloride (LiCl) were used in the production of dsRNAs and therefore fed to the bees together with the dsRNA, we chose to feed LiCl in sucrose solution to caged bees to test its activity against *Varroa* mites. Strikingly, LiCl at concentrations of 25 mM, which corresponds to the calculated concentration in the dsRNA solution, killed the mites as effectively as the test substances containing dsRNA. Moreover, after LiCl was largely removed from dsRNA by extensive washing (washed dsGFP ctrl), the miticidal activity was substantially diminished as indicated by delayed onset and reduced activity (Supplementary Fig. S1). From these data, we concluded that LiCl, not RNA knockdown, mediated the observed activity on *Varroa* mites and that it would be worthwhile to analyse the potential of LiCl as a varroacide.

### Effective concentration of lithium chloride

**Figure 1:**
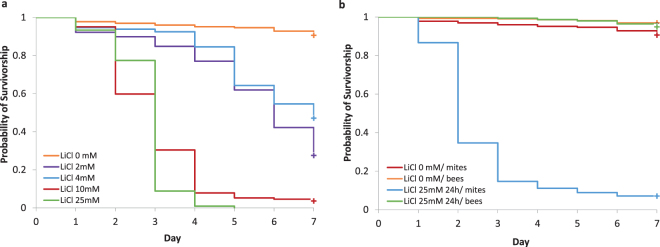
Mortality of phoretic *Varroa* mites and honey bees after feeding lithium chloride (LiCl) to caged bees. (**a**) Kaplan-Meier survival curve of female *Varroa* mites kept on caged bees fed with LiCl in concentrations between 2 mM–25 mM (n = 33, 9, 9, 12, and 9 cages for 0 mM (control), 2 mM, 4 mM, 10 mM and 25 mM, respectively). At all concentrations, the survival of mites in the treatment groups was significantly different from the control (*P* < 0.001, log-rank test with Bonferroni correction). (**b**) Kaplan-Meier survival curve of caged worker bees and female *Varroa* mites after 24 h LiCl exposure (n = 9 cages). The survival of mites in the treatment group was significantly different from the control group (*P* < 0.001, log-rank test) but there were no significant group differences in bee mortality.

To corroborate the primary observations of our pilot study, we established cage experiments with different concentrations of LiCl for robust statistical analysis. In addition to the 25 mM concentration, which was found to be effective in the pilot study, we used concentrations of 2 mM, 4 mM and 10 mM in order to determine the lower threshold of efficacy. The results supported the findings of the previous study and demonstrated significant miticidal effects for LiCl concentrations as low as 2 mM at which a substantial increase in mite mortality (*P* < 0.001, log-rank test; Supplementary Table S2) was shown. Higher concentrations of 10 mM and 25 mM both significantly enhanced mite mortality starting at day two of treatment and achieved extermination of more than 96% of the treated mites at the end of the experiment (Fig. 1a, Supplementary Table S2). In the control experiments without LiCl in the feeding solution, mite mortality reached on average 9.3% and was therefore well within the range of mortality rates obtained for mites kept on untreated cage bees under different environmental conditions^33^. Based on these results, we confirmed a clear effect of LiCl on mite viability in a concentration range between 2 mM and 25 mM.

In these experiments, the caged bees were fed with the respective concentration of LiCl over several days until all mites were killed by the treatment. However, for potential use in beekeeping practice, a shorter and defined treatment period would be preferable. We therefore performed an additional experiment, in which the most effective concentration of 25 mM LiCl (Fig. 1a) was administered for 24 h followed by feeding with sugar solution for additional six days. At the end of the observation period, 92.9% of the mites (n = 225 mites, *P* < 0.001, log-rank test) were killed without any significant effect on the treated bees (see next paragraph). This result clearly demonstrates that even a short-term feeding of 25 mM LiCl is sufficient to substantially diminish the mite population.

**Figure 2:**
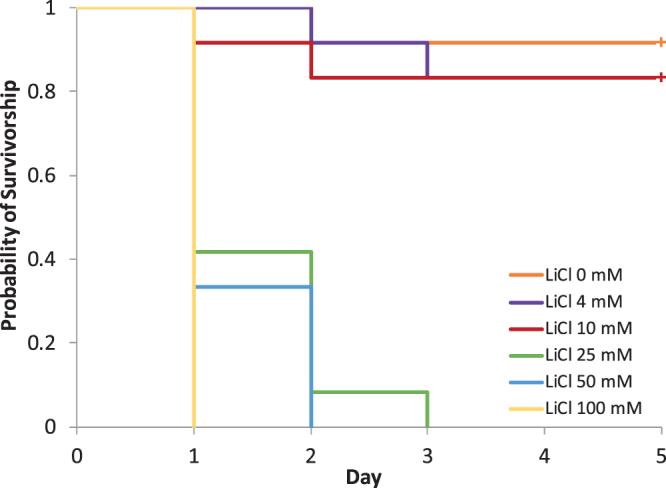
Mortality of phoretic *Varroa* mites kept on bees that were individually fed 10 µl of a lithium chloride solution at concentrations ranging from 4 mM to 100 mM. The bees were fed LiCl only once at the beginning of the experiment, then they received sucrose syrup over five days. For each concentration, 12 cages with one bee and one *Varroa* mite each were analysed. The survival of mites was significantly reduced compared to the control group when concentrations of 25 mM and higher were fed to the bees (*P* < 0.001, log-rank test).

To precisely determine the ingested amount of LiCl by the bees that is necessary to kill parasitizing mites, 12 newly hatched bees were artificially fed 10 µl of LiCl solutions of 4 mM to 100 mM and kept individually with a phoretic mite for five days within cages. With the 4 mM and 10 mM solutions, which corresponded to an uptake of 1.7 µg and 4.2 µg of LiCl, respectively, the effect was not significantly different from the untreated control (n = 12 mites, *P* = 1.000, log-rank test, Supplementary Table S3). However, a single dose of 25 mM, corresponding to 10.6 µg of LiCl consumed by the bee was sufficient to kill 100% of the phoretic mites within 48 hours (Fig. 2).

### Effect on worker bees

For the analysis of the tolerability of LiCl to worker bees, the test cages that were used to analyse mite mortality (Fig. 1a) were additionally recorded for the mortality of the worker bees. After exposure to 2 mM, 10 mM and 25 mM LiCl which have been shown to exert miticidal activity, the treated worker bee mortality ranged on average from 3 to 7% within the different feeding groups. With the exception of the 10 mM LiCl group (n = 12 cages, *P* = 0.015, log-rank test; Supplementary Table S4), the values were not significantly different from the 4% mortality in the untreated control group. Furthermore, the mortality rates of our controls were well within the range of the mortality of non-treated cage bees required as a control in toxicology tests^34^, therefore confirming the validity of our test system. Also the 24 h treatment with LiCl did not affect the mortality of worker bees (Fig. 1b; n = 9 cages, *P* = 0.308, log-rank test). A good tolerability of LiCl to bees was also confirmed by the feeding of a single dose (for mite mortality see Fig. 2) that did not elicit a significant increase in worker bee mortality (*P* = 1.000, log-rank test; Supplementary Table S5).

**Figure 3:**
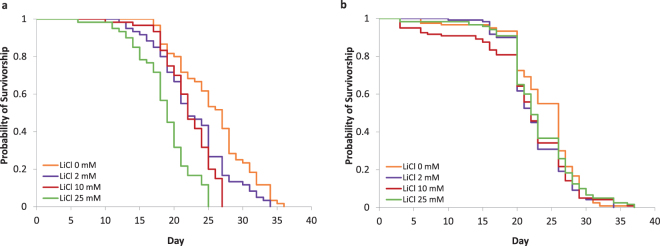
Mortality of honey bees after feeding lithium chloride to caged bees. (**a**) Kaplan-Meier survival curve of caged worker bees during chronic LiCl exposure. LiCl diets at concentrations of 2 mM, 10 mM and 25 mM were fed ad libitum till the death of the last bee (n = 6 cages with 10 bees each). The survival of all treated groups was significantly different from the sugar syrup control (*P* *<* 0.01, log-rank test with Bonferroni correction). (**b**) Kaplan-Meier survival curve of caged worker bees after a 24 h LiCl exposure. LiCl diets at concentrations of 2 mM, 10 mM and 25 mM were fed ad libitum for the first 24 h after hatching and then replaced by sucrose syrup (n = 12 cages with 10 bees each). The survival of all treated groups was not significantly different from the sugar syrup control (*P* > 0.1, log-rank test with Bonferroni correction).

Next, different concentrations of LiCl were continuously fed until the last caged bee died to investigate response to long-term exposure. Here, the treatment significantly reduced the average lifespan of freshly hatched worker bees from 26 days in the untreated control cages to 23 and 22 days for 2 mM and 10 mM LiCl, respectively (n = 60 bees, *P* = 0.024, log-rank test; Supplementary Table S6). In bees that received the highest concentration of 25 mM LiCl the lifespan was significantly reduced to 19 days on average (Fig. 3a).

However, LiCl appears to impede bee viability only if administered over an extended period of time as indicated by an additional experiment in which LiCl was fed for the first 24 h after hatching and was then replaced by sucrose syrup until the last caged bee died (Fig. 3b). Here, the average lifespan of freshly hatched worker bees ranged from 22 days (10 mM) to 24 days (control) without significant differences between the treatments (n = 120 bees per treatment, *P* ≥ 0.126, log-rank test; Supplementary Table S7). Based on this data from caged bees, we conclude that even a short-term LiCl treatment is sufficient to completely eradicate *Varroa* mite infestation with little or no impact on the viability of worker bees. These results obtained in cage tests under controlled conditions represent a successful and promising first step towards a new approach to *Varroa* treatment. However, efficiency and side effects must be confirmed under field conditions.

### Field tests with lithium chloride in artificial swarms

To approximate field conditions, we tested 25 mM and 50 mM LiCl in nine brood-free artificial swarms consisting of a queen and approximately 20,000 bees each. These concentrations were chosen based on previous experiments with caged bees using the highest dose that was still tolerated by bees in short application times (25 mM). Because an even distribution of sucrose syrup throughout the entire artificial swarm of approximately 20,000 bees might have been difficult to achieve, we additionally tested a 50 mM concentration of LiCl to ensure that each bee was exposed to sufficient amounts of lithium. Accordingly, the swarms were fed *ad libitum* with sucrose syrup containing 25 mM LiCl (n = 6) or 50 mM (n = 3) for a period of three days, followed by a topical application of Perizin®. Perizin® containing the organophosphate coumaphos as active ingredient, is a highly effective varroacide that is commonly used as a control treatment^35^. Mite mortality was monitored over a period of five days. Prior to the control treatment, 25 mM LiCl killed approximately 90% of the mites present within the artificial swarms (Table 1). The higher concentrated solution (50 mM), however, did not increase this effect (χ2 test, *P* = 0.953). Altogether, the efficacy was somewhat lower compared to the cage tests. One explanation might be that the distribution of LiCl within a cluster of thousands of bees requires more time until the last individual bee consumes a sufficient dosage to kill the respective parasitizing mite. The necessary feeding time of such huge entities of 20,000 bees and more must be analysed in further experiments.

**Table 1:** Comparison of the varroacidal action of two lithium chloride diets administered to artificial swarms for five days.

Lithium chloride concentration	Mite fall [Mean ± SD]			
	Swarms [n]	LiCl treatment	Perizin treatment	Efficacy [%]
25 mM	6	562.5 ± 149.4	65.5 ± 26.9	**88.9**
50 mM	3	178.3 ± 49.7	22.3 ± 3.1	**89.6**

Presented is the mean mite fall after the lithium chloride and final Perizin treatments and the calculated efficacy of the lithium treatment. The differences between the two lithium chloride treatments were not significant (χ2 test, *P* = 0.953).

### Efficacy of other lithium compounds and non-lithium salts

To confirm lithium as the active component for the effect on *Varroa* mites we tested a series of lithium compounds and compared the miticidal effects with non-lithium salts. Of particular interest were lithium citrate, a compound with three lithium ions, lithium sulphate and lithium carbonate, which have two lithium ions compared to only one lithium ion in LiCl. Additional compounds with one lithium ion (lithium lactate, lithium acetate), but different solubility, chemical reactivity and price were included to analyse efficacy and tolerability in comparison to LiCl. In cage experiments all compounds eliminated 100% of the mites at 25 mM within three (lithium citrate and lithium acetate) to four days (lithium sulphate, lithium lactate and lithium carbonate). Also, the 4-mM test solutions, entirely killed phoretic mites within five (lithium citrate, lithium sulphate, and lithium acetate) to seven days (lithium lactate) except for lithium carbonate (Table 2; Supplementary Table S8).

**Table 2:** Mortality of phoretic *Varroa* mites and worker bees after feeding two concentrations of different lithium compounds over a maximum feeding period of seven days.

	Control	Lithium sulphate		Lithium lactate		Lithium acetate		Lithium citrate		Lithium carbonate	
		4 mM	25 mM	4 mM	25 mM	4 mM	25 mM	4 mM	25 mM	4 mM	25 mM
Cages (n)	33	3	3	3	6	3	3	3	3	3	3
Mite mortality (% ± SD)	9.4 (±6.2)	100	100	100	100	100	100	100	100	94.7 (±9.2)	100
Bee mortality (% ± SD)	3.3 (±4.2)	4 (±2)	12.7 (±7)	4.7 (±2.3)	10 (±3.5)	9.3 (±4.2)	5.3 (±5.8)	5.6 (±6.9)	6 (±6)	2	3.3 (±4.2)

Worker bee mortality was not significantly increased at either concentration compared to the untreated control bees, except for 25 mM lithium sulphate and 25 mM lithium lactate (Supplementary Table S9). With these experiments we could confirm that other lithium compounds have a similar potential for the use as a systemic acaricide. This might increase the flexibility for the possible design of a veterinary product. Considering the price, lithium chloride and lithium citrate are the cheapest compounds. Lithium sulphate is less suitable due to the lower bee tolerability and lithium carbonate due to a relatively low water solubility.

To investigate the concentration-dependent efficacy of lithium compounds in more detail, we compared LiCl with lithium citrate (Li_3_C_6_H_5_O_7_), which had the greatest difference in the number of lithium ions per molecule, at five different concentrations in a range of 1 mM–25 mM. All concentrations of lithium citrate exhibited significantly higher acaricidal activity compared to LiCl, but there was no difference in the mortality of the bees (Table 3, Supplementary Tables S9 and S11). Therefore, lithium citrate might represent an even better active ingredient.

**Table 3:** Comparison of the efficacy and side effects of LiCl and lithium citrate using concentrations from 1 mM to 25 mM over a maximum feeding period of seven days.

Concentration	Lithium chloride			Lithium citrate		
	Cages (n)	Bee mortality (%)	Mite mortality (%)	Cages (n)	Bee mortality (%)	Mite mortality (%)
1 mM	6	1	29	3	3	48
2 mM	9	3	72	6	4	96
4 mM	9	4	53	6	6	100
10 mM	12	7	96	6	4	100
25 mM	9	5	100	3	6	100

As a lithium-free control and to rule out chloride as an active agent we also tested the alkaline salts sodium chloride (NaCl) and potassium chloride (KCl) and also magnesium chloride (MgCl) at 25 mM. We did not observe a varroacidal effect for NaCl or KCl (n = 3 cages, P = 1.000, log-rank test, Supplementary Table S12). In tests with MgCl 100% of the caged bees died within five days (P < 0.001, log-rank test), and according to the declining number of bees, the experiment was terminated before the effect on mites could be analysed. Based on these experiments, we concluded that lithium indeed mediates acaricidal activity in a dose-dependent manner and that lithium citrate exhibits the most favourable properties of all compounds tested so far.

### Potential of lithium compounds as new varroacide

We have shown that not the initially hypothesized double stranded RNAs against essential *Varroa* genes but surprisingly lithium salts mediate a strong acaricidal effect on *Varroa* mites on caged bees and in artificial swarms. Thus, these results show that lithium compounds represent a new class of acaricidal agents with an outstanding potential and remarkably good tolerability by bees. The different susceptibility of mites and bees to LiCl is even more remarkable considering that due to dilution effects, the concentration of LiCl in the haemolymph of the bees will probably be substantially lower than the concentration fed to the bees.

Importantly, our findings do not imply that acaricidal effects of RNAi-based approaches as published by Garbian *et al*.^32^ are generally mediated by LiCl. After feeding of a mixture of dsRNA to honey bees over a period of 60 days, Garbian *et al*.^32^ recorded a slow increase in mite mortality with a final treatment efficacy of only 60%. In view of the quick and highly effective response of our artificial swarms to treatments with LiCl, different modes of action are likely: while RNAi mediated effects appear to exert long-term effects, lithium compounds represent an independent mechanism with fast onset and high efficacy.

As a varroacide, LiCl displays some features that are unique in this combination: (i) LiCl acts systemically via honey bee feeding (“easy-to-apply”), (ii) it is water soluble and will therefore not accumulate in beeswax which is a crucial problem for long-term treatment concepts using synthetic varroacides with lipophilic properties^36,37^ (iii) the oral toxicity of most lithium compounds to mammals is relatively low^38^ (iv) it has no repellent effect on the feeding solution within the relevant concentration range of 2–25 mM^39^ and (v) it is available at moderate prices. Highly promising is the fact that a single application of only 10 µl of LiCl in a 25 mM solution (corresponding to a dosage of 10.6 µg of LiCl) per individual bee is sufficient to kill phoretic mites. A challenge for further research will be the development of a smart application technique for full-sized swarms and colonies to ensure that all bees receive the critical amount of the active compound.

Currently, we do not know how LiCl is killing the phoretic *Varroa* mites, and there are few publications on the effect of LiCl in insects^40^. In human medicine, lithium has been used since the 1870s and is a mood-stabilizing agent indicated for the treatment of manic episodes and as maintenance treatment for bipolar disorder^41^. In view of their therapeutic use, lithium compounds and their toxicity profile have been carefully investigated. Thus far, a number of enzymes acting on metabolism, development, haematopoiesis and other processes have been proposed as potential targets^42,43^. These enzymes require metal ions and lithium exerts its activity in an uncompetitive manner, which most likely occurs by displacing a divalent cation. Admittedly, we have currently no indication that the observed miticidal effect of lithium compounds relies on a comparable mode of action.

We are also aware of the fact that our results represent only the first step towards the development of a new veterinary product. Field tests in free-flying colonies are just as necessary as analysis of sublethal and long-term side effects on adult bees and honey bee brood and possible residue problems in honey.

However, the results presented here already indicate that LiCl has potential as an effective and easy-to-apply treatment for artificial and natural swarms and particularly for the huge number of package bees used for pollination in the United States^11,44^. Furthermore, elucidation of the mechanism of action might open new avenues for the targeted development of veterinary products to combat *Varroa* mites.

## Methods

### General configuration of the cage tests

The effect of lithium chloride, other lithium salts and control salts on *Varroa* mites feeding on treated bees was evaluated in cage tests^45^ consisting of 600-ml plastic containers (Lock and Lock*®*). A wax foundation (9 cm × 3.5 cm) was attached in the middle of the container and a hole for a 10-ml feeding syringe (Injekt®, Roth) was drilled into the bottom (Supplementary Fig. S2). The cage was turned around and closed with tights instead of the plastic lid. Longevity and single dose feeding experiments were performed in 40-ml containers (Rotilabo®, Roth) equipped with tights and 10 ml feeding syringes (Injekt®, Roth). The honey bees originated from colonies with low *Varroa* infestation levels and were collected from brood combs containing L5 (fifth larval instar) bee larvae. For the experiments on the total life span of worker bees and for the single dose application, freshly hatched bees from brood combs kept in an incubator were used. Female *Varroa* mites were collected from heavily infested colonies using the icing sugar method^46^, washed with lukewarm water and dried before transferring onto the caged bees. As soon as all cages were equipped with mites and honey bees, the feeding syringes were filled with the test or control feeding solution (Apiinvert®, Südzucker; dry substance content: 30% saccharose, 31% glucose, 39% fructose) and the cages were stored in a dark incubator (Memmert, 27 °C, 60% RH). Dead individuals were counted and removed daily, then the feeding solution was replaced.

### Pilot study: From RNAi to lithium chloride

In a first step, we used bioinformatics to identify essential *Varroa* genes. Sequences derived from published *Varroa destructor* contigs were blasted against the annotated genomes of *Drosophila melanogaster* and *Caenorhabditis elegans*. In a subsequent step sequences were selected that were shown to be essential in these organisms. Based on homology to these essential sequences, we selected 12 genes (Rpn1, Rpn3, Noi, GC2807, RPL7, ATP syn-beta, AlphaTub 84B, His2B, Med, RPL15, Blw and So) in the *Varroa* genome with high probability to impact mite viability after RNAi-mediated knockdown (Supplementary Table S1).

To obtain RNA, *Varroa* mites were frozen in liquid nitrogen and homogenized using a mortar and pestle. The RNA was isolated using Tri-Reagenz (Sigma Aldrich) according to the manufacturer’s instructions. Approximately 1 µg of RNA was used for cDNA synthesis (BioRad iScript Kit) in 20-µl reaction volume, and 0.5 µl of cDNA was used for the PCR reaction. Each PCR was performed with *Varroa* gene-specific primers with an additional T7 polymerase primer sequence at the 5’ end to allow T7 RNA polymerase-dependent RNA transcription. For each gene, two PCRs were performed: one with a T7 primer site for the sense strand and one with a T7 primer site for the antisense strand. This step was done to enable strand-specific *in vitro* transcription of each gene. After *in vitro* transcription, the RNA was precipitated using 2.5 M final concentration of LiCl. The supernatant was completely removed, and the RNA was dissolved in RNAse-free water. The sense and antisense RNA transcripts were mixed together in equimolar concentrations and annealed by heating 5 min at 95 °C followed by slowly cooling down to ~50 °C. The annealed RNA (dsRNA) was then ready to use in the cage tests.

Per cage, 100 honey bees were infected with 30 *Varroa* mites and fed with sugar syrup containing 0.8 µg of dsRNA per gene, bee and day. Accordingly, a total of 960 µg of dsRNA was fed daily. Besides sugar syrup, a similar amount of dsRNA based on the coding sequence for the green fluorescent protein GFP (dsGFP ctrl) served as a control (n = 3 cages each). In successive experiments sugar syrup containing washed dsGFP (n = 1 cage) and LiCl at a concentration of 25 mM (n = 2 cages) was fed to the bees. The bees received the respective diet for the whole observation period of four days.

### Effective concentration of lithium chloride

The acaricidal effect of lithium chloride (Roth ≥ 99%) was evaluated by feeding of five concentrations of a sugar syrup/lithium chloride solution ranging from 1 mM to 25 mM to infested honey bees (n = 6, 9, 9, 12, and 9 cages for 1 mM, 2 mM, 4 mM, 10 mM and 25 mM, respectively). Per cage 50 (±2) honey bees and 25 (±2) *Varroa* mites were used. As soon as all mites died the test solution was substituted with sugar syrup. After seven days, the remaining bees and mites were freeze-killed and counted. The experimental period of seven days was here used because with the two most effective concentrations of 10 mM and 25 mM already all mites died within this time period and we did not intend to test the effects of a chronic application on mite mortality. For a long-term survival test of worker bees longer time periods were used (see below). For a comparison of LiCl with a compound with three lithium ions, lithium citrate (Roth ≥ 98% Ph. Eur) was tested in the same concentration range (n = 3, 6, 6, 6, and 3 cages for 1 mM, 2 mM, 4 mM, 10 mM and 25 mM, respectively).

We further tested the effect of a short-term treatment by feeding 25 mM lithium chloride for 24 hours only (n = 9 cages). Afterwards, the feeding solution with lithium chloride was replaced by Apiinvert® for six more days.

To define the critical dosage that effectively kills parasitizing *Varroa* mites, newly hatched honey bees were individually fed after a starving period of 3 hours with either 10 µl of sugar syrup or 10 µl of a lithium chloride/ sucrose solution ranging from 4 mM to 100 mM. Per concentration 12 honey bees were fed and kept individually. Each honey bee was infested with one phoretic *Varroa* mite directly after hand feeding and received a sugar syrup diet during the following observation period of five days.

### Effect on worker bees

The longevity of honey bees fed lithium chloride was tested in separate cage tests containing either sugar syrup or a lithium chloride solution at concentrations of 2 mM, 10 mM and 25 mM (n = 6 cages each). Each cage contained 10 freshly hatched honey bees that were constantly fed with the respective solution until all of the bees died.

### Field tests with lithium chloride in artificial swarms

To approximate natural conditions, we tested the acaricidal activity of lithium chloride in brood-free artificial swarms^47^. Therefore, 9 artificial swarms with approximately 2 kg of honey bees each and a caged queen were placed in closed boxes and kept at 15 °C in the dark. After being established, the artificial swarms were fed sucrose syrup with either 25 mM (n = 6) or 50 mM (n = 3) lithium chloride via a container for three days. After the treatment, all swarms were transferred from the boxes to hives at an apiary and allowed to forage. Food was provided ad libitum and mite mortality was monitored on a daily base. The remaining mites were removed using 50 ml of Perizin® per swarm by trickling and subsequent counting of mites over a period of two days.

### Efficacy of other lithium compounds and non-lithium salts

We further tested lithium citrate (Roth ≥ 98% Ph. Eur), lithium sulphate (Roth ≥ 99%), lithium carbonate (Roth ≥ 99%), lithium acetate (Roth ≥ 99%) and lithium lactate (Sigma Aldrich 95%) at concentrations of 4 and 25 mM (n = 3 cages each). In the control tests, the caged bees were fed with Apiinvert® only (n = 33 cages). As an additional control, magnesium chloride (Roth ≥ 98,5%) and the alkali salts potassium chloride (Roth ≥ 99%) and sodium chloride (Roth ≥ 99%) were fed in the same manner at a concentration of 25 mM (n = 3 cages each). For detailed comparison with LiCl, lithium citrate was tested at additional concentrations. All cage tests were performed in the same way as described under “Effective concentration of lithium chloride”.

### Statistics

The data sets obtained from cage tests were analysed using *SPSS* 22 statistics software. A survival distribution was estimated from the continuous death times of *Varroa* mites and honey bees by performing a Kaplan-Meier survival estimate. Survivorship between treatments was compared pairwise and tested for significance with log-rank tests followed by a Bonferroni correction. A chi-squared test was used to compare mite fall data of artificial swarms treated with two different lithium chloride concentrations. Differences between groups with *P* < 0.05 were considered statistically significant.

## Electronic supplementary material

Supplementary material
