# Author Correction: Lithium chloride effectively kills the honey bee parasite *Varroa destructor* by a systemic mode of action

**DOI:** 10.1038/s41598-018-21311-2

**Published:** 2018-03-06

**Authors:** Bettina Ziegelmann, Elisabeth Abele, Stefan Hannus, Michaela Beitzinger, Stefan Berg, Peter Rosenkranz

**Affiliations:** 1 University of Hohenheim, Apicultural State Institute, 70593 Stuttgart, Germany; 2 siTOOLs Biotech GmbH, Lochhamerstrasse 29A, 82152 Planegg, Germany; 3 Bayerische Landesanstalt für Weinbau und Gartenbau, Fachzentrum Bienen, An der Steige 15, 97209 Veitshöchheim, Germany

Correction to: *Scientific Reports* 10.1038/s41598-017-19137-5, published online 12 January 2017

The Acknowledgements section in this Article was omitted. The Acknowledgements section should read:

“This work has been supported by the Bavarian Research Foundation (AZ-1200-15)”.

