# Supplementary material for: Lithium chloride effectively kills the honey bee parasite Varroa destructor by a systemic mode of action
Source: Sci Rep. 2018 Jan 12;8:683. doi: 10.1038/s41598-017-19137-5 (PMC5766531; doi:10.1038/s41598-017-19137-5)
Supplement: Supplementary file 1 — Supplementary material [file 41598_2017_19137_MOESM1_ESM.doc]

**Lithium chloride effectively kills the honey bee parasite *Varroa destructor* by a systemic mode of action**

***Bettina Ziegelmann*1*, Elisabeth Abele*1*, Stefan Hannus*2*, Michaela Beitzinger*2*, Stefan Berg*3*, Peter Rosenkranz*1**

**1** University of Hohenheim, Apicultural State Institute. 70593 Stuttgart, Germany.

**2** siTOOLs Biotech GmbH, Lochhamerstrasse 29A. 82152 Planegg, Germany.

**3** Bayerische Landesanstalt für Weinbau und Gartenbau, Fachzentrum Bienen, An der Steige

15. 7209 Veitshöchheim, Germany.

**Corresponding author:** Bettina Ziegelmann; bettina.ziegelmann@uni-hohenheim.de

**Supplementary Table S1. List of potentially essential *Varroa* genes. *Varroa* sequence contigs were first blasted against the *D. melanogaster* genome to identify homologous genes. Identified genes were run against the online gene essentiality database (OGEE_db) to identify genes essential in *Drosophila*, *C. elegans*, zebrafish (*D. erio*) and human. The table indicates the identified *Varroa* sequence contigs and the corresponding Fly gene symbols, the NCBI ID and its description. The last column shows in which organisms the gene has been shown to be essential.**

|  | **Varroa sequence Contigs** | **Fly symbol** | **Fly NCBI Gen ID** | **Description** | **Species where essential** |
| --- | --- | --- | --- | --- | --- |
| **1** | VDK00094244-1167_3 [1135 - 20] (REVERSE SENSE) | Rpn1 | 40174 | Regulatory particle non-ATPase 1 | fly,human,worm |
| **2** | VDK00000139-9843_8 [879 - 106] (REVERSE SENSE) | Rpn3 | 35176 | Regulatory particle non-ATPase 3 | fly,human,worm |
| **3** | VDK00043141-2197_2 [1551 - 181] (REVERSE SENSE) | noi | 40678 | noisette | fly,worm,zebrafish |
| **4** | VDK00009960-4164_6 [3327 - 4163] | CG2807 | 33235 | CG2807 gene product from transcript CG2807-RB | fly,worm,zebrafish |
| **5** | VDK00027265-2807_2 [1458 - 1946] | RpL7 | 34352 | Ribosomal protein L7 | fly,worm,zebrafish |
| **6** | VDK00075236-1459_1 [180 - 890] | ATPsyn-beta | 43829 | ATP synthase-beta | fly,worm |
| **7** | VDK00095194-1154_2 [1027 - 347] (REVERSE SENSE) | Tbp-1 | 42805 | Tat-binding protein-1 | fly,worm |
| **8** | VDK00073905-1481_1 [3 - 1133] | alphaTub84B | 40848 | alpha-Tubulin at 84B | fly,worm |
| **9** | VDK00071883-1518_1 [848 - 1516] | Rpt4 | 31567 | Regulatory particle triple-A ATPase 4 | fly,human,zebrafish |
| **10** | VDK00108999-979_1 [977 - 132] (REVERSE SENSE) | Pros26.4 | 42828 | Proteasome 26S subunit subunit 4 ATPase | fly,human |
| **11** | VDK00082374-1341_1 [810 - 1340] | PP2A-B' | 42169 | CG7913 gene product from transcript CG7913-RK | fly,human |
| **12** | VDK00090385-1223_4 [569 - 33] (REVERSE SENSE) | RpL15 | 3354918 | Ribosomal protein L15 | fly,human |
| **13** | VDK00011255-3993_4 [3992 - 3195] (REVERSE SENSE) | Rpn2 | 43449 | Regulatory particle non-ATPase 2 | fly |
| **14** | VDK00027501-2796_1 [348 - 1328] | blw | 37617 | bellwether | fly |
| **15** | VDK00042688-2211_5 [1125 - 334] (REVERSE SENSE) | so | 35662 | sine oculis | fly |
|  |  |  |  |  |  |

**Supplementary Table S2.** Statistical comparison of the survivorship of *Varroa* mites parasitizing on caged honey bees that were fed ad libitum with different concentrations of lithium chloride diets. The bees were fed with the lithium chloride diet until all mites had died followed by sucrose syrup. Per cage 50 bees and 25 mites were treated and observed for seven days.

Presented are the *P* values of a pairwise comparison of the survivorship of *Varroa* mites (Log-rank test followed by a Bonferroni correction).

*P* values marked with asterisks were considered statistically significant with ****P* < 0.001.

| **Lithium chloride concentration** | **Significance** | | | | |
| --- | --- | --- | --- | --- | --- |
|  | **Cages [n]** | **2 mM** | **4 mM** | **10 mM** | **25 mM** |
| **0 mM** | 33 | < 0.001*** | < 0.001*** | < 0.001*** | < 0.001*** |
| **2 mM** | 9 |  | < 0.001*** | < 0.001*** | < 0.001*** |
| **4 mM** | 9 |  |  | < 0.001*** | < 0.001*** |
| **10 mM** | 12 |  |  |  | 0.230 |
| **25 mM** | 9 |  |  |  |  |

**Supplementary Table S3.** Statistical comparison of the survivorship of *Varroa* mites parasitizing on honey bees individually fed with 10 µl of a lithium chloride solution containing a definite amount of lithium chloride at the beginning of the experiment. The bees were then fed with sucrose syrup and the mortality of mites and bees was observed for five days. Each cage contained one mite and one newly hatched honey bee.

| **Lithium chloride concentration** | **Amount of LiCl fed to the bee [µg]** |  | **Significance** | | | | |
| --- | --- | --- | --- | --- | --- | --- | --- |
|  |  | **Cages [n]** | **4 mM** | **10 mM** | **25 mM** | **50 mM** | **100 mM** |
| **0 mM** | 0 | 12 | 1.000 | 1.000 | < 0.001*** | < 0.001*** | < 0.001*** |
| **4 mM** | 1.7 | 12 |  | 1.000 | < 0.001*** | < 0.001*** | < 0.001*** |
| **10 mM** | 4.2 | 12 |  |  | < 0.001*** | < 0.001*** | < 0.001*** |
| **25 mM** | 10.6 | 12 |  |  |  | 1.000 | 0.208 |
| **50 mM** | 21.2 | 12 |  |  |  |  | 0.480 |
| **100 mM** | 42.4 | 12 |  |  |  |  |  |

Presented are the *P* values of a pairwise comparison of the survivorship of *Varroa* mites (Log-rank test followed by a Bonferroni correction). *P* values marked with asterisks were considered statistically significant with ****P* < 0.001.

**Supplementary Table S4.** Statistical comparison of the survivorship of caged honey bees fed ad libitum with different lithium chloride diets. The bees were fed with the lithium chloride diet until all mites had died followed by sucrose syrup or, in case that not all mites died, for seven days until the end of the experiment. Per cage 50 bees and 25 mites were treated and observed for seven days.

Presented are the *P* values of a pairwise comparison of the survivorship of honey bees (Log-rank test followed by a Bonferroni correction). *P* values marked with asterisks were considered statistically significant with **P* < 0.05.

| **Lithium chloride concentration** | **Significance** | | | | |
| --- | --- | --- | --- | --- | --- |
|  | **Cages [n]** | **2 mM** | **4 mM** | **10 mM** | **25 mM** |
| **0 mM** | 33 | 1.000 | 1.000 | 0.015* | 1.000 |
| **2 mM** | 9 |  | 1.000 | 0.010* | 1.000 |
| **4 mM** | 9 |  |  | 0.227 | 1.000 |
| **10 mM** | 12 |  |  |  | 0.834 |
| **25 mM** | 9 |  |  |  |  |

| **Lithium chloride concentration** | **Amount of LiCl fed to the bee [µg]** |  | **Significance** | | | | |
| --- | --- | --- | --- | --- | --- | --- | --- |
|  |  | **Cages [n]** | **4 mM** | **10 mM** | **25 mM** | **50 mM** | **100 mM** |
| **0 mM** | 0 | 12 | 1.000 | 1.000 | 1.000 | 1.000 | 1.000 |
| **4 mM** | 1.7 | 12 |  | 1.000 | 1.000 | 1.000 | 1.000 |
| **10 mM** | 4.2 | 12 |  |  | 1.000 | 1.000 | 1.000 |
| **25 mM** | 10.6 | 12 |  |  |  | 1.000 | 1.000 |
| **50 mM** | 21.2 | 12 |  |  |  |  | 1.000 |
| **100 mM** | 42.4 | 12 |  |  |  |  |  |

**Supplementary Table S5.** Statistical comparison of the survivorship of honey bees individually fed with 10 µl of a lithium chloride solution containing a definite amount of lithium chloride at the beginning of the experiment. The bees were then fed with sucrose syrup and the mortality of mites and bees was observed for five days. Each cage contained one mite and one newly hatched honey bee.

Presented are the *P* values of a pairwise comparison of the survivorship of honey bees (Log-rank test followed by a Bonferroni correction).

**Supplementary Table S6.** Statistical comparison of the survivorship of caged honey bees which were fed ad libitum with different concentrations of lithium chloride from hatching until death. Each cage contained ten newly hatched honey bees.

Presented are the *P* values of a pairwise comparison of the survivorship of honey bees (Log-rank test followed by a Bonferroni correction). *P* values marked with asterisks were considered statistically significant with **P* < 0.05 and ****P* < 0.001.

| **Lithium chloride concentration** | **Significance** | | | |
| --- | --- | --- | --- | --- |
|  | **Cages [n]** | **2 mM** | **10 mM** | **25 mM** |
| **0 mM** | 6 | 0.024* | < 0.001*** | < 0.001*** |
| **2 mM** | 6 |  | 0.522 | < 0.001*** |
| **10 mM** | 6 |  |  | < 0.001*** |
| **25 mM** | 6 |  |  |  |

**Supplementary Table S7.** Statistical comparison of the survivorship of caged honey bees which were fed ad libitum with different concentrations of lithium chloride diets for 24h right after hatching followed by sucrose syrup until death. Each cage contained ten newly hatched honey bees.

Presented are the *P* values of a pairwise comparison of the survivorship of honey bees (Log-rank test followed by a Bonferroni correction).

| **Lithium chloride concentration** | **Significance** | | | |
| --- | --- | --- | --- | --- |
|  | **Cages [n]** | **2 mM** | **10 mM** | **25 mM** |
| **0 mM** | 12 | 0.126 | 0.278 | 1.000 |
| **2 mM** | 12 |  | 1.000 | 1.000 |
| **10 mM** | 12 |  |  | 1.000 |
| **25 mM** | 12 |  |  |  |

**Supplementary Table S8.** Statistical comparison of the survivorship of *Varroa* mites parasitizing on caged honey bees that were fed ad libitum with different lithium salt diets. The bees were fed with the lithium salt diet until all mites had died followed by sucrose syrup. Per cage 50 bees and 25 mites were treated and observed for seven days.

Presented are the *P* values of a pairwise comparison of the survivorship of *Varroa* mites (Log-rank test followed by a Bonferroni correction). *P* values marked with asterisks were considered statistically significant with ****P* < 0.001.

| **Diet** | **Concentration** |  | | |  | | | | | | | **Significance** | | |  | | | |  | |  |
| --- | --- | --- | --- | --- | --- | --- | --- | --- | --- | --- | --- | --- | --- | --- | --- | --- | --- | --- | --- | --- | --- |
|  |  |  | | **Lithium sulphate** | | |  | | **Lithium citrate** | |  | | **Lithium acetate** | |  | **Lithium lactate** | |  | | **Lithium carbonate** | |
|  |  | **Cages [n]** | **4 mM** | | | **25 mM** | | **4 mM** | | **25 mM** | | **4 mM** | | **25 mM** | **4 mM** | | **25 mM** | | **4 mM** | | **25 mM** |
| **Sucrose syrup** | 0 mM | 33 | <0.001*** | | | <0.001*** | | <0.001*** | | <0.001*** | | <0.001*** | | <0.001*** | <0.001*** | | <0.001*** | | <0.001*** | | <0.001*** |
| **Lithium sulphate** | 4 mM | 3 |  | | | <0.001*** | | <0.001*** | | <0.001*** | | <0.001*** | | <0.001*** | <0.001*** | | <0.001*** | | <0.001*** | | <0.001*** |
|  | 25 mM | 3 |  | | |  | | <0.001*** | | 0.032 | | <0.001*** | | 1.000 | <0.001*** | | 0.351 | | <0.001*** | | <0.001*** |
| **Lithium citrate** | 4 mM | 3 |  | | |  | |  | | <0.001*** | | 1.000 | | <0.001*** | <0.001*** | | <0.001*** | | <0.001*** | | 0.815 |
|  | 25 mM | 3 |  | | |  | |  | |  | | <0.001*** | | 1.000 | <0.001*** | | 1.000 | | <0.001*** | | 0.001*** |
| **Lithium acetate** | 4 mM | 3 |  | | |  | |  | |  | |  | | <0.001*** | <0.001*** | | <0.001*** | | <0.001*** | | 0.234 |
|  | 25 mM | 3 |  | | |  | |  | |  | |  | |  | <0.001*** | | 1.000 | | <0.001*** | | <0.001*** |
| **Lithium lactate** | 4 mM | 3 |  | | |  | |  | |  | |  | |  |  | | <0.001*** | | 1.000 | | <0.001*** |
|  | 25 mM | 3 |  | | |  | |  | |  | |  | |  |  | |  | | <0.001*** | | 0.125 |
| **Lithium carbonate** | 4 mM | 3 |  | | |  | |  | |  | |  | |  |  | |  | |  | | <0.001*** |
|  | 25 mM | 3 |  | | |  | |  | |  | |  | |  |  | |  | |  | |  |

**Supplementary Table S9.** Statistical comparison of the survivorship of caged honey bees that were fed ad libitum with different lithium salt diets. The bees were fed with the lithium salt diet until all mites had died followed by sucrose syrup. Per cage 50 bees and 25 mites were treated and observed for seven days.

Presented are the *P* values of a pairwise comparison of the survivorship of honey bees (Log-rank test followed by a Bonferroni correction). *P* values marked with asterisks were considered statistically significant with **P*<0.05 and ****P* < 0.001.

| **Diet** | **Concentration** |  | | |  | | | | | | | **Significance** | | |  | | | |  | |  |
| --- | --- | --- | --- | --- | --- | --- | --- | --- | --- | --- | --- | --- | --- | --- | --- | --- | --- | --- | --- | --- | --- |
|  |  |  | | **Lithium sulphate** | | |  | | **Lithium citrate** | |  | | **Lithium acetate** | |  | **Lithium lactate** | |  | | **Lithium carbonate** | |
|  |  | **Cages [n]** | **4 mM** | | | **25 mM** | | **4 mM** | | **25 mM** | | **4 mM** | | **25 mM** | **4 mM** | | **25 mM** | | **4 mM** | | **25 mM** |
| **Sucrose syrup** | 0 mM | 33 | 1.000 | | | <0.001*** | | 1.000 | | 1.000 | | 0.206 | | 1.000 | 1.000 | | 0.036* | | 1.000 | | 1.000 |
| **Lithium sulphate** | 4 mM | 3 |  | | | 0.320 | | 1.000 | | 1.000 | | 1.000 | | 1.000 | 1.000 | | 1.000 | | 1.000 | | 1.000 |
|  | 25 mM | 3 |  | | |  | | 0.475 | | 1.000 | | 1.000 | | 1.000 | 0.830 | | 1.000 | | 0.020* | | 0.164 |
| **Lithium citrate** | 4 mM | 3 |  | | |  | |  | | 1.000 | | 1.000 | | 1.000 | 1.000 | | 1.000 | | 1.000 | | 1.000 |
|  | 25 mM | 3 |  | | |  | |  | |  | | 1.000 | | 1.000 | 1.000 | | 1.000 | | 1.000 | | 1.000 |
| **Lithium acetate** | 4 mM | 3 |  | | |  | |  | |  | |  | | 1.000 | 1.000 | | 1.000 | | 0.391 | | 1.000 |
|  | 25 mM | 3 |  | | |  | |  | |  | |  | |  | 1.000 | | 1.000 | | 1.000 | | 1.000 |
| **Lithium lactate** | 4 mM | 3 |  | | |  | |  | |  | |  | |  |  | | 1.000 | | 1.000 | | 1.000 |
|  | 25 mM | 3 |  | | |  | |  | |  | |  | |  |  | |  | | 0.185 | | 1.000 |
| **Lithium carbonate** | 4 mM | 3 |  | | |  | |  | |  | |  | |  |  | |  | |  | | 1.000 |
|  | 25 mM | 3 |  | | |  | |  | |  | |  | |  |  | |  | |  | |  |

**Supplementary Table S10.** Statistical comparison of the survivorship of *Varroa* mites parasitizing on caged honey bees that were fed ad libitum with different concentrations of lithium chloride and lithium citrate diets respectively. The bees were fed with the lithium salt diet until all mites had died followed by sucrose syrup. Per cage 50 bees and 25 mites were treated and observed for seven days.

Presented are the *P* values of a pairwise comparison of the survivorship of *Varroa* mites (Log-rank test followed by a Bonferroni correction).

*P* values marked with asterisks were considered statistically significant with **P*<0.05*,* ***P*<0.005 and ****P* < 0.001.

| **Diet** | **Concentration** |  |  |  |  | | **Significance** | | |  | | |  |  |
| --- | --- | --- | --- | --- | --- | --- | --- | --- | --- | --- | --- | --- | --- | --- |
|  |  |  | **Lithium chloride** | | | | |  | **Lithium citrate** | | | | | |
|  |  | **Cages [n]** | **1 mM** | **2 mM** | **4mM** | **10 mM** | **25 mM** | **1 mM** | | **2 mM** | **4 mM** | **10 mM** | | **25 mM** |
|  | 0 mM | 33 | <0.001*** | <0.001*** | <0.001*** | <0.001*** | <0.001*** | <0.001*** | | <0.001*** | <0.001*** | <0.001*** | | <0.001*** |
| **Lithium chloride** | 1 mM | 6 |  | <0.001*** | <0.001*** | <0.001*** | <0.001*** | 0.245 | | <0.001*** | <0.001*** | <0.001*** | | <0.001*** |
|  | 2 mM | 9 |  |  | <0.001*** | <0.001*** | <0.001*** | 0.031* | | <0.001*** | <0.001*** | <0.001*** | | <0.001*** |
|  | 4 mM | 9 |  |  |  | <0.001*** | <0.001*** | 1.000 | | <0.001*** | <0.001*** | <0.001*** | | <0.001*** |
|  | 10 mM | 12 |  |  |  |  | 1.000 | <0.001*** | | <0.001*** | 1.000 | <0.001*** | | 0.015* |
|  | 25 mM | 9 |  |  |  |  |  | <0.001*** | | <0.001*** | 0.002** | <0.001*** | | 0.020* |
| **Lithium citrate** | 1 mM | 3 |  |  |  |  |  |  | | <0.001*** | <0.001*** | <0.001*** | | <0.001*** |
|  | 2 mM | 6 |  |  |  |  |  |  | |  | <0.001*** | <0.001*** | | <0.001*** |
|  | 4 mM | 6 |  |  |  |  |  |  | |  |  | <0.001*** | | <0.001*** |
|  | 10 mM | 6 |  |  |  |  |  |  | |  |  |  | | 0.184 |
|  | 25 mM | 3 |  |  |  |  |  |  | |  |  |  | |  |

**Supplementary Table S11.** Statistical comparison of the survivorship of caged honey bees that were fed ad libitum with different concentrations of lithium chloride and lithium citrate diets respectively. The bees were fed with the lithium salt diet until all mites had died followed by sucrose syrup. Per cage 50 bees and 25 mites were treated and observed for seven days.

Presented are the *P* values of a pairwise comparison of the survivorship of honey bees (Log-rank test followed by a Bonferroni correction).

*P* values marked with asterisks were considered statistically significant with ***P*<0.005.

| **Diet** | **Concentration** |  |  |  |  | | **Significance** | | |  | | |  |  |
| --- | --- | --- | --- | --- | --- | --- | --- | --- | --- | --- | --- | --- | --- | --- |
|  |  |  | **Lithium chloride** | | | | |  | **Lithium citrate** | | | | | |
|  |  | **Cages [n]** | **1 mM** | **2 mM** | **4mM** | **10 mM** | **25 mM** | **1 mM** | | **2 mM** | **4 mM** | **10 mM** | | **25 mM** |
|  | 0 mM | 33 | 0.542 | 1.000 | 1.000 | 0.081 | 1.000 | 1.000 | | 1.000 | 1.000 | 1.000 | | 1.000 |
| **Lithium chloride** | 1 mM | 6 |  | 1.000 | 0.838 | 0.003** | 0.288 | 1.000 | | 1.000 | 0.083 | 0.978 | | 0.105 |
|  | 2 mM | 9 |  |  | 1.000 | 0.053 | 1.000 | 1.000 | | 1.000 | 1.000 | 1.000 | | 1.000 |
|  | 4 mM | 9 |  |  |  | 1.000 | 1.000 | 1.000 | | 1.000 | 1.000 | 1.000 | | 1.000 |
|  | 10 mM | 12 |  |  |  |  | 1.000 | 1.000 | | 1.000 | 1.000 | 1.000 | | 1.000 |
|  | 25 mM | 9 |  |  |  |  |  | 1.000 | | 1.000 | 1.000 | 1.000 | | 1.000 |
| **Lithium citrate** | 1 mM | 3 |  |  |  |  |  |  | | 1.000 | 1.000 | 1.000 | | 1.000 |
|  | 2 mM | 6 |  |  |  |  |  |  | |  | 1.000 | 1.000 | | 1.000 |
|  | 4 mM | 6 |  |  |  |  |  |  | |  |  | 1.000 | | 1.000 |
|  | 10 mM | 6 |  |  |  |  |  |  | |  |  |  | | 1.000 |
|  | 25 mM | 3 |  |  |  |  |  |  | |  |  |  | |  |

**Supplementary Table S12.** Mortality rates and statistical comparison of the survivorship of caged honey bees and parasitizing *Varroa* mites. The bees were fed ad libitum with sucrose solutions containing either sodium chloride, potassium chloride or magnesium chloride. Each cage contained 25 mites and 50 honey bees and was observed for 7 days.

Presented are the *P* values of a pairwise comparison of the survivorship of *Varroa* mites (Log-rank test followed by a Bonferroni correction) and honey bees, respectively. The P values of the mite mortality for MgCl treatment could not be calculated as all bees died within five days and the experiment discontinued. *P* values marked with asterisks were considered statistically significant with ***P*<0.005 and ****P* < 0.001.

| **Diet** |  | | **Significance** | | |  | |  | **Significance** | | |  |
| --- | --- | --- | --- | --- | --- | --- | --- | --- | --- | --- | --- | --- |
|  | **Cages [n]** | **Bee mortality [%** ±SD**]** | **NaCl** | **KCl** | **MgCl** | | **Mite mortality [%** ±SD **]** | **NaCl** | | **KCL** | **MgCl** | |
| Sucrose syrup | 33 | 3.3 (± 4.2) | 1.000 | 0.001** | < 0.001*** | | 9.4 (± 6.2) | 1.000 | | 1.000 | - | |
| NaCl 25 mM | 3 | 3 (± 3.1) |  | 0.070 | < 0.001*** | | 9 (± 5.8) |  | | 1.000 | - | |
| KCl 25 mM | 3 | 11 (± 1.2) |  |  | < 0.001*** | | 11 (± 2.3) |  | |  | - | |
| MgCl 25 mM | 3 | 100 |  |  | | | 27 (± 2.3) |  | |  |  | |

**Supplementary Figure S1**: Mortality of *Varro*a mites kept on caged bees that were fed with different diets. *Varro*a specific dsRNA, unspecific dsGFP-RNA and LiCl as the precipitating agent were fed to caged bees and compared to sugar syrup as untreated control. As an additional control, dsGFP-RNA after additional washing steps to remove LiCl was used.


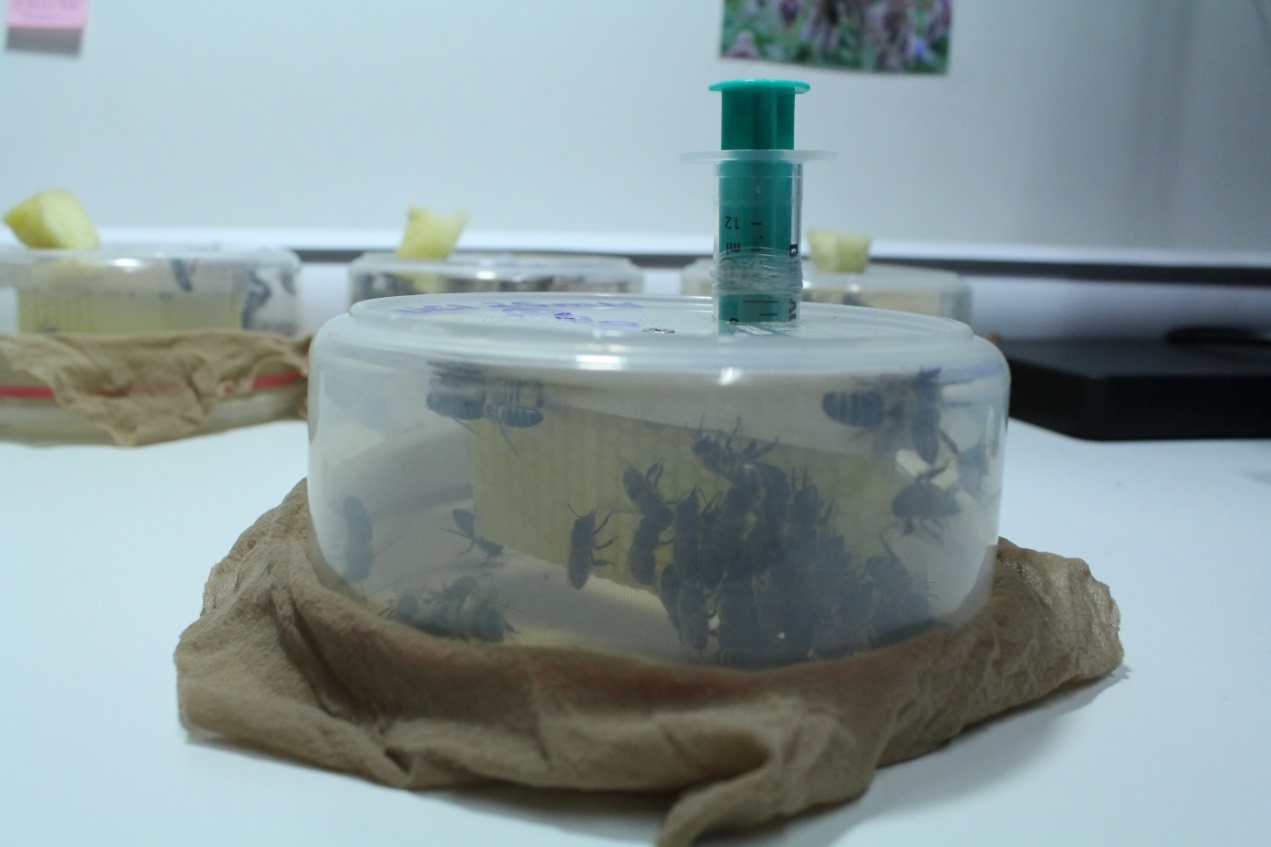


**Supplementary Figure S2**: Plastic cage with a piece of a wax comb foundation and a syringe for ad libitum feeding of different concentrations of lithium compounds and non-lithium salts. For each mite-survival test up to 50 bees and 25 phoretic *Varroa* mites (bee:mite-ratio 2:1, except for the preliminary tests) were kept in an incubator for a period of 7 days. The experiments on the longevity of bees lasted over longer time periods until all worker bees died.
